# Supplementary material for: In Vitro Induction of Trained Innate Immunity by bIgG and Whey Protein Extracts
Source: Int J Mol Sci. 2020 Nov 28;21(23):9077. doi: 10.3390/ijms21239077 (PMC7731221; doi:10.3390/ijms21239077)
Supplement: Supplementary file 1 [file ijms-21-09077-s001.pdf]

Supplementary Materials

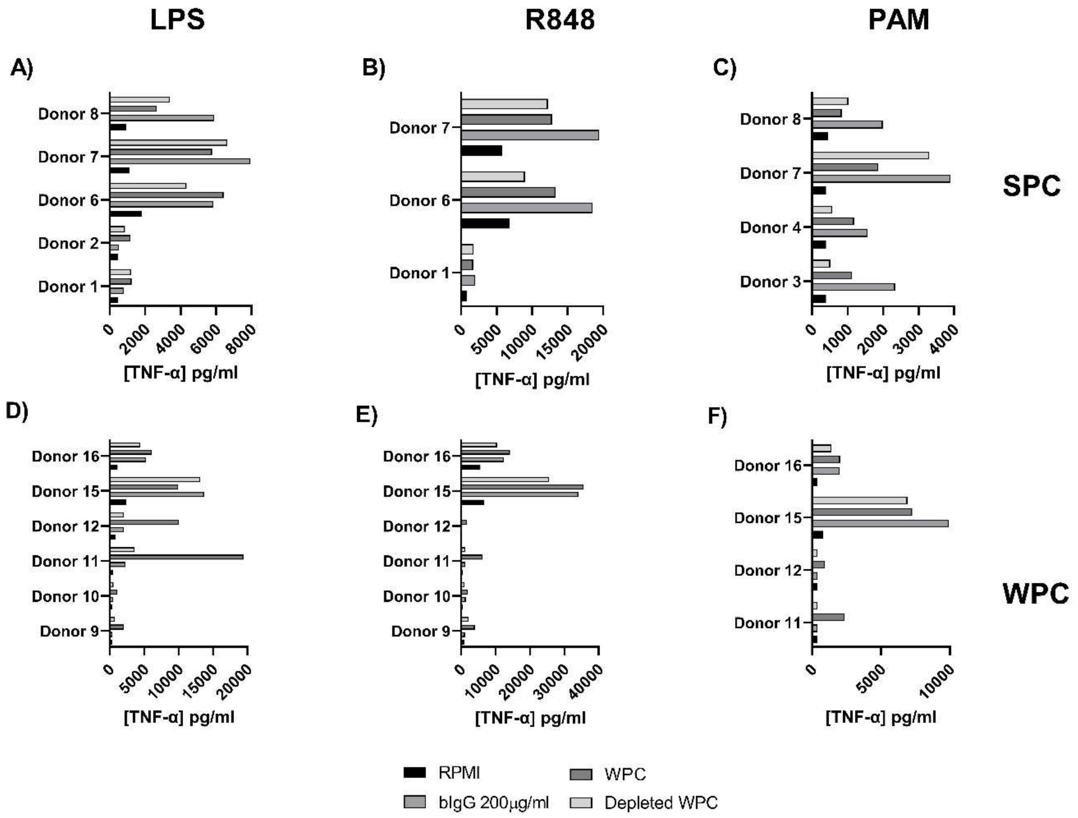

Supplementary Figure 1. Effect of depletion of serum protein concentrate (SPC) and whey protein concentrate (WPC) from bIgG on the induction of trained immunity per SPC- and WPC-trained donor. Monocytes were isolated from fresh buffy coats and after allowing the monocytes to adhere for 2 hours, the training stimuli depleted SPC (diluted eq. to 200µg bIgG/ml non-depleted SPC), depleted WPC (diluted eq. to 200µg bIgG/ml non-depleted WPC) and the negative control RPMI. After 24h incubation with the stimulus, the monocytes were washed to remove the stimuli. On day 6, the monocytes were re-stimulated with (**a,d**) LPS (100ng/ml), (**b,e**) R848 (10µg/ml) or (**c,f**) PAM (Pam3CSK4, 10µg/ml). After 24h incubation, supernatant was collected for assessment of (**a-c**) TNF-α and (**e-f**) IL-6 production. Only donors for which the TNF-α production after re-stimulation was more than 50% higher for SPC or WPC training, compared to RPMI, were included in this figure. For each individual donor and treatment, the percentage of TNF-α production of training with depleted SPC or WPC and control was determined compared to training with non-depleted SPC or WPC, respectively. The data is shown as mean ± standard deviation of the donors meeting the requirement for inclusion.

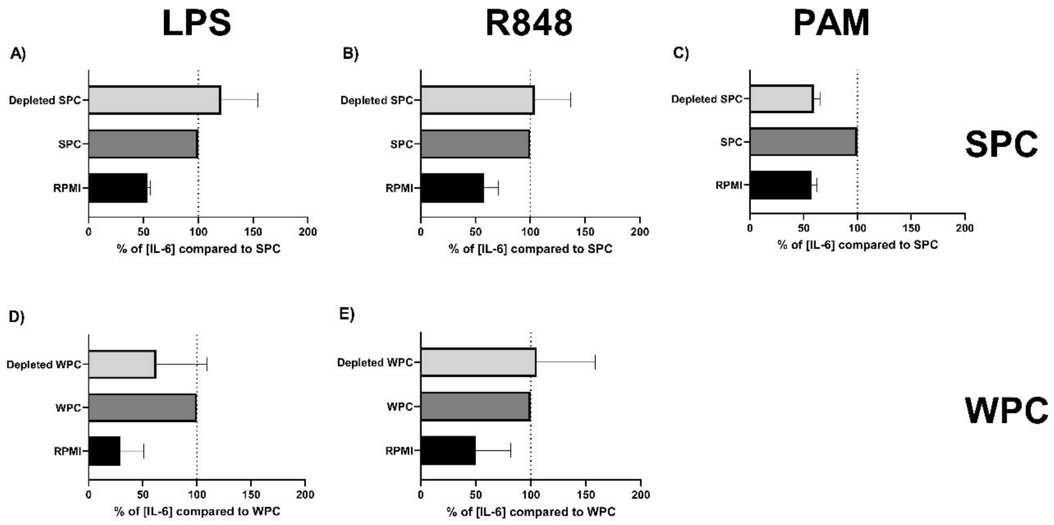

Supplementary Figure 2. Effect of depletion of bIgG from SPC and WPC on the induction of trained immunity in primary monocytes. Monocytes were isolated from fresh buffy coats and after allowing the monocytes to adhere for 2 hours, the training stimuli depleted SPC (diluted eq. to 200μg bIgG/ml non-depleted SPC), depleted WPC (diluted eq. to 200μg bIgG/ml non-depleted WPC) and the negative control RPMI. After 24h incubation with the stimulus, the monocytes were washed to remove the stimuli. On day 6, the monocytes were re-stimulated with (**a,d**) LPS (100ng/ml), (**b,e**) R848 (10μg/ml) or (**c**) PAM (Pam3CSK4, 10μg/ml). After 24h incubation, supernatant was collected and IL-6 production was measured. Data are shown as % IL-6 production relative to the non-depleted SPC and WPC. Error bars represent the standard deviation.
